# Interactions of periodontal pathogens with megakaryocytic cells and platelets

**DOI:** 10.1080/20002297.2017.1325245

**Published:** 2017-06-09

**Authors:** A.M. Andrews, S. Haywood-Small, T. Smith, P. Stafford

**Affiliations:** ^a^ Biomolecular Sciences Research Centre, Sheffield Hallam University, Sheffield, UK

## Abstract

**Introduction**: Cardiovascular disease (CVD) is a leading cause of morbidity, accounting for around 17.3 million deaths worldwide. Recent studies have linked periodontitis to CVD with the periodonto-pathogens *Porphyromonas gingivalis* and *Tannerella forsythia* thought to contribute and exacerbate atherosclerosis through interactions with platelets. To date, while platelet activation following challenge with periodonto-pathogens has been reported, the underlying mechanisms of these interactions are yet to be elucidated. The aim of this study is to determine how periodonto-pathogens interact with platelets using both megakaryocytic cells and isolated platelets.

**Methods**: To characterise expression levels of surface markers including ubiquitously expressed platelet-specific markers (CD41, CD42b) and platelet activation markers (CD62P, PAC-1), a multi-colour flow cytometry panel was developed using undifferentiated megakaryocytic cells CHRF-288-11 before validation using platelets isolated from healthy donors. Changes in levels of surface markers following bacterial challenge both with megakaryocytic cells and isolated platelets were determined using flow cytometry. Interaction with pathogens was visualised by platelet aggregometry and fluorescence microscopy using pathogen-specific antibodies.

**Results and conclusions**: Both pathogens invaded megakaryocytic cells as visualised by immunofluorescence microscopy. The pathogens also bound platelets causing increased levels of aggregation and upregulated expression of activation markers including in CD62P in flow cytometric assays.

